# Sleep Disturbance as a Mediator Between Problematic Social Media Use and Depressive Symptoms Among Mexican Undergraduate Nursing Students: A Multicenter Study

**DOI:** 10.3390/ejihpe15110229

**Published:** 2025-11-11

**Authors:** José Ángel Hernández-Mariano, Ana Cristina Castañeda-Márquez, Gledy Manuela Olmos-Rivera, Rocío Castillo-Díaz, Fani Villa-Rivas, Edith Araceli Cano-Estrada, Yaneth Citlalli Orbe-Orihuela, Miguel Trujillo-Martínez, Monica Alethia Cureño-Díaz

**Affiliations:** 1 Department of Research, Hospital Juarez of Mexico, Mexico City 07760, Mexico; 2 Scientific Research Institute, Juarez University of the State of Durango, Durango 34000, Mexico; 3 Nursing School, Hospital Juárez de México, Mexico City 04510, Mexico; 4 Faculty of Nursing and Midwifery, Juarez University of the State of Durango, Durango 34217, Mexico; 5 Superior School of Tlahuelilpan, Autonomous University of the State of Hidalgo, Tlahuelilpan 42780, Mexico; 6 Department of Chronic Infections and Cancer, Center for Research on Infectious Diseases, National Institute of Public Health, Cuernavaca 62100, Mexico; 7 General Hospital with Family Medicine Unit Number 7, Mexican Social Security Institute, Cuautla 62740, Mexico; 8 Department of Institutional Intelligence in Oncological Health, National Institute of Cancerology, Mexico City 14080, Mexico

**Keywords:** social media addiction, depression, sleep disorders, nursing students

## Abstract

Background: Social media addiction is a growing concern among undergraduates, with nursing students particularly vulnerable as their stressful academic and clinical context may promote excessive use, impaired sleep, and higher depression risk. Therefore, we aimed to evaluate whether sleep disturbance mediates the association between problematic social media use and symptoms of depression among nursing students in Mexico. Methods: We conducted a multicenter, cross-sectional, analytical study using a random sample of 638 nursing students from three Mexican public universities. Between August and December 2024, participants completed validated scales for social media addiction, sleep quality, and depression. Counterfactual causal mediation analysis was performed using logistic regression with robust errors. Results: Sleep disturbance affected 51.7% of students, and 27.5% reported depression. Social media addiction showed a dose–response association with both outcomes. Mediation analysis revealed a total effect on depression (OR = 2.19; 95% CI = 1.45–3.30) and an indirect effect via sleep disturbance (OR = 1.22; 95% CI = 1.01–1.38), explaining 31.4% of the effect. Conclusions: Sleep disturbance partially mediates this association. Interventions addressing digital behavior and sleep hygiene may improve mental health in nursing students.

## 1. Introduction

Mental health disorders represent a significant challenge to global public health due to their high prevalence and profound impact. It is estimated that approximately one in eight individuals worldwide lives with a mental disorder (Fan et al., 2025), with depression being the most prevalent. Recent estimates suggest a global prevalence of depressive disorders of 3588.25 cases per 100,000 population (Zhang et al., 2024). In Mexico, it has been reported that 16% of young adults present with depressive symptoms, most of whom do not receive adequate care (Vázquez-Salas et al., 2023). This situation highlights the structural deficiencies of the mental health system, primarily due to the shortage of specialized services and the inequitable distribution of community and outpatient resources nationwide (Carmona-Huerta et al., 2021).

In this sense, undergraduate students are particularly vulnerable to developing mental disorders due to their constant exposure to academically and emotionally challenging environments. Factors such as academic overload, pressure to meet institutional expectations, peer competition, performance anxiety, and personal difficulties can act as chronic stressors that negatively affect their mental health (Liu et al., 2023; Mofatteh, 2020).

Among them, nursing students may be especially at risk of experiencing psychological distress due to the nature of their training. In addition to acquiring theoretical and technical knowledge, they are required to engage in clinical practice from the early stages of their education, often in emotionally intense and high-pressure environments. These include providing care for critically ill patients, witnessing human suffering, and facing death (Labrague et al., 2018; Silva et al., 2011). The emotional intensity of these experiences, which are uncommon in most other academic programs, may increase their susceptibility to anxiety, stress, burnout, and depressive symptoms (Consuelos-Sánchez et al., 2023; Hernández-Mariano et al., 2024; Sonmez et al., 2023; Vo et al., 2023). A recent meta-analysis estimated that, globally, approximately three in ten nursing students (29%) experience depression (Efstathiou et al., 2025).

Although depressive symptoms are also prevalent among students from other academic disciplines, nursing students face distinctive emotional and professional demands that differentiate them from the general college population (Akhtar et al., 2020). From the early stages of training, they are exposed to ethically challenging and emotionally charged situations linked to patient care—conditions rarely encountered in other academic programs. These experiences, combined with academic overload, irregular schedules, and clinical stress, may heighten their vulnerability to maladaptive coping behaviors, including excessive social media use and sleep disruption. Therefore, nursing students should not be regarded merely as representative of all university students but rather as a particularly sensitive subgroup within the health sciences, whose experiences may resemble those of other care-oriented disciplines such as medicine, psychology, or social work.

Currently, the study of emerging factors that could influence the mental health of undergraduate students has gained relevance, particularly the intensive use of social media (Salari et al., 2025). Social media addiction has been defined as a pattern of compulsive, uncontrolled, and persistent use of these platforms, which interferes with daily activities and generates significant distress when access is limited or interrupted (Amirthalingam & Khera, 2024). Although the evidence is still emerging, some studies suggest that this behavior could be related to sleep disorders (O. Ahmed et al., 2024).

Sleep disorders, defined as any alteration in the quantity, quality, or continuity of nighttime rest that compromises physical or psychological functioning during the day (Karna et al., 2025), have been associated with prolonged social media use; this association is particularly significant when social media use is concentrated at night (O. Ahmed et al., 2024; Alonzo et al., 2021). It has been suggested that factors such as screen time before bed, sustained emotional arousal, and difficulty mentally disconnecting from the digital environment may contribute to delayed sleep onset, frequent nighttime interruptions, or daytime sleepiness (Allen et al., 2018; Blume et al., 2019; Dresp-Langley & Hutt, 2022; Kinsella & Chin, 2024; Kumar et al., 2025). Nonetheless, the mechanisms underlying this possible relationship are not yet fully understood and may vary across individuals and contexts.

On the other hand, it has also been suggested that social media addiction could be associated with a higher likelihood of experiencing depressive symptoms. While several explanatory mechanisms have been proposed, such as negative social comparison, low self-esteem, or overexposure to emotionally intense content (M. Ahmed, 2023; Firth et al., 2024; Lițan, 2025; Samra et al., 2022), sleep impairment has been suggested as a possible mediator in this relationship. According to this hypothesis, problematic social media use may impair sleep quality, which in turn could disrupt neurotransmitter regulation and promote the secretion of proinflammatory cytokines, both mechanisms that have been implicated in the pathophysiology of depression (Dziurkowska & Wesolowski, 2021; Nicolaides et al., 2000; Nishuty et al., 2019). This interaction is especially relevant in university settings with high academic demands and unstable sleep routines.

Among nursing students, these conditions may be further exacerbated by the dual demands of theoretical instruction and practical clinical training, as well as by early exposure to emotionally taxing healthcare environments (Labrague et al., 2018; Silva et al., 2011). This issue becomes even more concerning given evidence suggesting that mental health problems that arise during undergraduate training often persist after students transition into the workforce (Rudman & Gustavsson, 2012). Nevertheless, few studies have simultaneously examined the relationship between social media addiction, sleep quality, and depressive symptoms in this specific population (AlAmer et al., 2020; Berdida, 2025). In the Mexican context, although these issues have been described individually (Bueno-Brito et al., 2024; Márquez-Ríos & Grimaldo-Zapata, 2024; Pérez et al., 2024; Vázquez et al., 2025), their interrelationships have been comparatively less explored. Investigating these associations may not only help to quantify the magnitude of the problem but also to identify potential targets for interventions aimed at promoting the well-being of future nursing professionals.

Recent global research has consistently demonstrated that excessive social media use is associated with sleep disturbances, depressive symptoms, and burnout among university students (Alimoradi et al., 2019; Corke et al., 2025; Osman, 2025; Yu et al., 2024). However, empirical studies examining these mechanisms in Latin American settings remain scarce, particularly among health sciences students. Although the biological and psychological mechanisms linking social media use, sleep disturbances, and depressive symptoms are considered universal, primarily involving circadian rhythm disruption, dopaminergic reward dysregulation, and the bidirectional relationship between sleep and mood (Levenson et al., 2017; Scott et al., 2021), their expression may vary across educational and social environments. In Mexico and other countries in the region, undergraduate students and clinical interns in health sciences represent an essential component of the healthcare workforce, often assuming patient care responsibilities during their training (Hernández-Mariano et al., 2024; Pan American Health Organization, 2025; World Health Organization, 2020).

This dual academic and clinical role exposes them to irregular schedules, emotional stress, and reduced sleep opportunities, conditions that may intensify the effects of problematic social media use on mental health. Expanding the evidence base in these populations is crucial to assess the external validity of current models and to design contextually relevant interventions that promote mental well-being among future healthcare professionals.

Therefore, we evaluated the association between problematic social media use, sleep disturbance, and depressive symptoms in undergraduate Mexican nursing students, testing a conceptual model in which sleep disturbance mediates the relationship between problematic social media use and depressive symptoms.

## 2. Materials and Methods

### 2.1. Design and Study Population

We conducted a multicenter, analytical, cross-sectional study among undergraduate nursing students in Mexico. The study population consisted of 1388 students enrolled in nursing training programs at three public universities located in Mexico City, Hidalgo, and Durango, between August and December 2024. The minimum sample size required for this study was calculated using the formula for relative measures of association (i.e., odds ratio) (Cvetković-Vega et al., 2021). We determined a sample size of 638 participants to estimate an odds ratio of at least 1.7, based on the following assumptions: a global prevalence of social media addiction among undergraduate students of 18.4% (Salari et al., 2025), a statistical power of 80%, and a 95% confidence level. Participants were selected using proportional stratified sampling by study site (Mexico City, Hidalgo, and Durango). Study participants were men and women aged 18 years or older. Pregnant women and students who were undergoing treatment with psychiatric medication at the time of the survey were excluded from the study.

### 2.2. Data Collection

General information was obtained through a structured questionnaire that included questions on the sociodemographic characteristics of the nursing students (i.e., sex, age, marital status, family monthly income, offspring, employment, and parental schooling); academic information (i.e., year in nursing program and scholarship); regular tobacco and alcohol consumption (i.e., current consumption greater than or equal to once per week); and we also obtained information on the frequency of social media utilization.

The problematic use of social media was assessed using the Social Media Addiction Scale-Student Form (SMAS-SF) (Sahin, 2018), which has been previously validated in the Mexican population (Valencia-Ortiz & Cabero-Almenara, 2019) and demonstrated acceptable internal consistency (Cronbach’s alpha = 0.93). The SMAS-SF consists of 29 items organized into four dimensions and rated on a five-point Likert scale. (0, hardly ever; 1; 2, Sometimes; 3, Almost always). The SMAS-SF’s scores range from a minimum of 29 to a maximum of 145. The ut-off points for classifying the level of problematic social media use are the following: 29–51, no addiction; 52–74, mild dependence; 75–97, moderate dependence; and >98, high dependency.

The Pittsburgh Sleep Quality Index (PSQI), which evaluates overall sleep quality over the past month, was used to assess sleep disturbance. The PSQI comprises 19 items grouped into seven subscales: self-reported sleep quality, sleep latency, sleep duration, habitual sleep efficiency, sleep disturbances, use of sleeping medication, and daytime dysfunction. Each of them is scored on a scale from 0 to 3, and the total global score is obtained by summing the component scores, yielding a range from 0 to 21. Higher scores indicate poorer sleep quality. A global PSQI score ≥ 5 indicates poor sleep. The PSQI has been previously validated in undergraduate students, demonstrating acceptable internal consistency (Cronbach’s alpha = 0.78) (Ojeda-Paredes et al., 2019).

For depressive symptoms screening, we employed the straightforward seven-item shortened version of the Center for Epidemiologic Studies Depression Scale (CESD-7). This version, previously validated in the Mexican population, shows acceptable internal consistency (Cronbach’s alpha > 0.83). The CESD-7 consists of 7 items and uses a 4-point Likert-type response scale. The total score, calculated by summing the item responses, ranges from 0 to 21. A score of 5 or higher indicates the presence of depression (Salinas-Rodríguez et al., 2014).

At each participating institution, students were invited through classroom announcements and institutional communication channels. Those who agreed to participate received detailed information about the study’s objectives, voluntary nature, and confidentiality safeguards. After providing written informed consent, participants completed the self-administered questionnaire in supervised classroom sessions to ensure standardized conditions and to prevent discussion among participants. The average response time was approximately 20 min. All questionnaires were collected anonymously and coded to preserve participant confidentiality.

### 2.3. Statistical Analysis

The study variables were described using frequencies and percentages for categorical variables. We compared the general characteristics of the participants according to their sleep disturbance and depression status using the Pearson Chi-square test. The associations between social media addiction, sleep disturbance, and depression were evaluated using logistic regression models for each independent variable. The dose–response relationships between interest variables were also assessed in all models (a trend value of *p* < 0.05 was considered significant) (Patino & Ferreira, 2016).

Because sleep disturbance is a known risk factor for mood deterioration, we explored its mediating effect in the pathway connecting problematic social media use with depressive symptoms. To facilitate this analysis, we collapsed the last three categories of the “social media addiction” variable, thereby dichotomizing it into two strata: “no addiction” and “some degree of addiction.” We then conducted a mediation analysis using binary exposure and mediator variables (Rijnhart et al., 2023). In our study, we utilized Stata’s “mediate” command, a key tool in the counterfactual mediation model (Hicks & Tingley, 2011). This model, also known as the potential outcomes approach, is instrumental in decomposing the total effect of an exposure on an outcome into its direct and indirect components through a mediator (Byeon & Lee, 2023). We specified logistic models for the mediator (sleep disturbance) and the outcome (depression), both dichotomous. To ensure the robustness of our findings, we conducted a sensitivity analysis using the bootstrap method with 1000 replications (Jung, 2021). The consistency of the results with those of the original model validated our approach, leading us to report the effects estimated from the “mediate” command, expressed as odds ratios (OR) using the “stat or” command. Furthermore, we presented the percentage each effect (direct and indirect) represents relative to the total effect, aiding interpretation of the relative magnitude of each pathway.

To assess the robustness of the findings and verify whether the classification of social media addiction influenced the mediation results, we conducted a sensitivity analysis using an alternative grouping of the exposure variable. In this additional model, the categories no addiction and mild addiction were combined into a single reference group, while moderate and severe addiction were merged to represent problematic use. This reclassification aimed to explore whether the observed associations were consistent when using a broader definition of social media addiction. The mediation analysis was repeated using this dichotomized classification, with the same counterfactual framework, model specifications, and bootstrap procedure described above.

All models were adjusted for confounding factors. The selection of confounders for inclusion in the models was based on directed acyclic graphs (DAGs) (Cañón-Montañez & Rodríguez-Acelas, 2019; Digitale et al., 2022; Tennant et al., 2021). Age, sex, year of study in the nursing program, paid work, family monthly income, study site, family history of mental illness, and parental education were identified as the minimal sufficient adjustment set for the association between social media addiction and depression (Supplementary Figure S1). Variables not selected by the DAGs were further evaluated as potential confounders using the change-in-estimate criterion, whereby a variable was considered a confounder if its inclusion in the model produced a meaningful change (≥10%) in the OR for the association between exposure and outcome. Alcohol and tobacco consumption were assessed using this approach; however, they were not included in the final models, as their inclusion did not yield meaningful changes in the effect estimates. Statistical significance for all logistic models was determined using a *p*-value threshold of <0.05. All analyses were conducted using Stata Statistical Software, version 19.5 (StataCorp, College Station, TX, USA).

## 3. Results

The characteristics of nursing students and their differences according to their sleep disturbance and depression status are summarized in Table 1. The participants’ ages ranged from 18 to 31 years, with women comprising 80.8% of the total. Most were single, had no children, were unemployed, had a family income of less than $470, were in their second year of university, and did not have a university scholarship. Furthermore, 12.1% of the participants reported consuming alcohol, and 10.2% reported using tobacco at least once per week. In addition, most participants reported spending 3–5 h per day on social media.

Overall, 51.7% of the participants showed signs of sleep disturbance, and 27.5% showed signs of depression. Among students with sleep disturbances, a higher proportion reported having offspring and spending between three and five hours per day on social media, compared to those with good sleep quality. Similarly, among participants with depression, a greater proportion had lower monthly household income and lower maternal educational attainment, relative to those without depression. Furthermore, the prevalence of depression increased among students who reported spending more time on social media.

The distribution of sleep disturbance and depressive symptoms across levels of problematic social media use is presented in Table 2. The prevalence of sleep problems increased steadily with greater dependence severity, ranging from 36.8% among non-dependent participants to 69.2% among those with high dependence. A similar gradient was observed for depressive symptoms.

After adjusting for potential confounders, students with mild social media dependence showed a higher, but not statistically significant, probability of reporting sleep disturbances compared with non-dependent peers (aOR = 1.31; 95% CI: 0.81–2.10). In contrast, those with moderate (aOR = 2.22; 95% CI: 1.37–3.61) and high dependence (aOR = 3.73; 95% CI: 2.19–6.35) had markedly increased odds of experiencing sleep problems. The magnitude of this association increased progressively across dependence categories, with substantially higher values in the moderate and high groups than in the mild group. Furthermore, a dose–response trend was observed between social media dependence and sleep disturbance (*p* for trend = 0.001; see Table 3).

No statistically significant association was found between mild social media dependence and depressive symptoms. In contrast, students with moderate (aOR = 2.56; 95% CI: 1.41–4.64) and high dependence (aOR = 4.54; 95% CI: 2.45–8.44) showed a significantly greater likelihood of presenting depressive symptoms compared with those without problematic social media use. The magnitude of this association increased progressively across dependence categories, and a clear dose–response trend was observed between social media use and depressive symptomatology (*p* for trend < 0.001; see Table 4).

**Figure 1:**
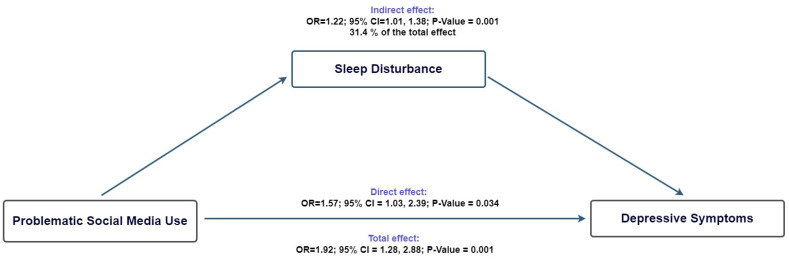
Counterfactual mediation model of problematic social media use, sleep disturbance, and depressive symptoms.

Figure 1 summarizes the results of the mediation analysis. Our findings indicate that the association between problematic social media use and depressive symptoms was partially mediated by sleep disturbance, which accounted for 31.4% of the total effect. The indirect pathway was statistically significant (adjusted odds ratio [aOR] = 1.22; 95% confidence interval [CI] = 1.01, 1.38), while the direct effect remained substantial (aOR = 1.57; 95% CI = 1.03, 2.39). These results suggest that, although sleep disturbance plays a mediating role, most of the association (68.6%) is likely explained by other mechanisms.

To evaluate the robustness of these findings, a sensitivity analysis was performed using an alternative definition of problematic social media use. In this model, participants with no or mild addiction were grouped as a single reference category, and those with moderate or severe addiction were classified as having problematic use. The mediation analysis under this dichotomized classification yielded a natural indirect effect of 1.21 (95% CI 1.07–1.36; *p*-value = 0.002) and a natural direct effect of 2.41 (95% CI 1.69–3.43; *p*-value = 0.001). The proportion mediated through sleep disturbance was 20% (95% CI 0.06–0.33). The consistency of these estimates with those of the main model supports the stability of the observed relationships (Supplementary Figure S2).

## 4. Discussion

Our findings revealed a significant association between problematic social media use and depressive symptoms, with sleep disturbances accounting for a modest but statistically significant proportion of this relationship.

In our study, 51.7% of participants reported poor sleep quality, a substantial proportion, though notably lower than the 80% prevalence reported in a recent study from Tamaulipas, Mexico, despite both using the same measurement tool (Pérez et al., 2024). This discrepancy may be explained by methodological differences, such as the smaller sample size in the previous study (n = 99), as well as by contextual and academic differences across institutions. Additionally, the limited set of covariates considered in that study may have restricted the exploration of other relevant psychosocial factors. In contrast, our multicenter design, spanning different regions of the country, provides a more comprehensive and representative perspective.

Moreover, compared with findings from the international literature, the prevalence of sleep disturbance observed in our study falls within the expected range, typically reported as 30–50% in similar student populations (Efstathiou et al., 2025; Ibrahim et al., 2024). This consistency supports the robustness of our estimates and reinforces the relevance of sleep disturbance as a common concern in this demographic group.

In our study, the prevalence of depressive symptoms was 27.5%. This estimate contrasts with findings from two recent studies conducted in Mexico. Márquez-Ríos and Grimaldo-Zapata (2024) reported a prevalence of 15.5%, whereas Bueno-Brito et al. (2024) documented a markedly higher prevalence of 52.36%. These discrepancies could be attributed to various methodological differences, such as the instrument used to assess depression and the smaller sample size, both of which were smaller than in our study. Furthermore, in the Bueno-Brito et al. (2024) study, the sample consisted exclusively of first-year students. The transition to university life represents a particularly challenging period, characterized by separation from family life, increased academic demands, and the need to assume new personal and professional responsibilities. These factors, combined, make the first year of university a particularly vulnerable time for mental health.

In the international context, a systematic review with meta-analysis reported an overall prevalence of depression of 29% in nursing students (Efstathiou et al., 2025), a figure closer to that observed in our study. However, it is essential to note that a few of the primary studies included in this meta-analysis were conducted in Latin American students; thus, the prevalence of this affective problem in the region may not be adequately represented. Despite this, the figures above reaffirm that depression constitutes a significant mental health problem among nursing students around the world.

Our findings showed that moderate and high levels of social media dependence were associated with a greater likelihood of sleep disturbance. The association followed a clear gradient, with higher dependence levels corresponding to increasingly frequent sleep problems, supporting a dose–response relationship between social media use and sleep quality. Our findings are consistent with several previous studies conducted in nursing students and the general university population, which have documented that addictive use of social media and smartphones is associated with altered sleep patterns (Haddaouy et al., 2025; Kalal et al., 2023; Rahman et al., 2025; Setyowati et al., 2023; Zhu et al., 2023).

Several underlying mechanisms could explain the observed association. During the night, in the absence of sunlight, the retina sends signals to the suprachiasmatic nucleus of the hypothalamus (considered the body’s biological clock), which stimulates the pineal gland to produce and release melatonin into the blood. This hormone primarily regulates the circadian rhythm, especially the sleep–wake cycle (Patel et al., 2025; Pevet et al., 2021). Nevertheless, exposure to artificial light, including that emitted by electronic devices such as tablets, computers, and mobile phones (commonly used for checking social media), can mimic natural sunlight, interfering with melatonin synthesis and consequently disrupting standard sleep patterns (Allen et al., 2018; Blume et al., 2019; Kumar et al., 2025). Likewise, constant interaction with social media, especially at night, can lead to cognitive activation, making it difficult to fall asleep and reducing its quality (Firth et al., 2019; Joshi, 2022). Furthermore, the highly stimulating or entertaining content often found on these platforms can increase alertness and prolong vigilance. On the other hand, stressful content, social comparison, and the pressure to stay connected can also generate anxiety or emotional distress, which in turn contributes to a greater vulnerability to sleep disorders (O. Ahmed et al., 2024; Korte, 2020).

Consistent with the previously described associations, we found that problematic social media use is significantly associated with higher odds of having depressive symptoms. This finding is consistent with reports from different studies, which have documented that problematic use of these platforms is linked to a negative impact on the mental health of nursing students and generally young people (Cai et al., 2021; David et al., 2024; Jiayuan et al., 2025; Osman, 2025).

In this context, compulsive social media use can interfere with daily routines, affect motivation for activities outside the digital environment, lead to difficulties in setting personal boundaries, and result in feelings of frustration, all of which contribute to a deterioration in emotional well-being (M. Ahmed, 2023; Firth et al., 2024). The intermittent feedback provided by these platforms (based on notifications, social interactions, and virtual rewards) could reinforce addictive behaviors and fuel negative emotional states when expectations are not met. This pattern of interaction can generate a vicious cycle in which excessive social media use becomes both a cause and a consequence of depression (J. Wang & Wang, 2025).

It is important to note that, in our analysis, mild social media dependence was not significantly associated with either depression or sleep disturbances. This may reflect frequent but non-dysfunctional use, which is common and often adaptive among university students, given the academic and social utility of these platforms. From a methodological standpoint, the instrument’s sensitivity to detect subtle impairments may be limited, and its classification thresholds may not clearly distinguish between intensive but functional use and early signs of problematic behavior. Additionally, the sample size within this category might have been insufficient to detect modest associations. Nonetheless, the significant trend across dependence levels supports a dose–response pattern, indicating that the adverse effects of social media use increase progressively with greater dependence intensity (Abi-Jaoude et al., 2020; Geng & Liu, 2025; Soraci et al., 2024; Yan et al., 2024).

Through our mediation analysis, we identified that sleep disturbances partially mediated the overall effect of problematic social media use on depressive symptoms. Specifically, a significant indirect effect through these disturbances was found, explaining approximately 31.4% of the total association.

This finding underscores the potential role of sleep disturbance as a biological and behavioral pathway linking problematic social media use to depression. Sleep disturbances can directly affect the neuroendocrine, immune systems, and neurotransmitter systems, all of which are implicated in the pathophysiology of depression. Partial or chronic sleep deprivation can disrupt the hypothalamic–pituitary–adrenal (HPA) axis, leading to increased cortisol secretion, a hormone closely associated with stress and depression (Dziurkowska & Wesolowski, 2021; Nicolaides et al., 2000). Sleep disruption is also linked to reduced availability of serotonin and dopamine (Moncrieff et al., 2023). Moreover, sleep fragmentation and decreased deep sleep have been linked to increased systemic inflammation, as reflected in elevated markers such as interleukin-6 (IL-6) and C-reactive protein, which have also been implicated in the etiology of depression (Nishuty et al., 2019).

On the other hand, sleep disorders have the potential to weaken a person’s coping strategies when dealing with daily stress (Palmer & Alfano, 2017). Lack of adequate rest impairs emotional self-regulation, which increases people’s vulnerability to experiencing intense negative emotions, such as depression, in the face of stressful events (Y. Wang et al., 2023). This may be especially relevant in the university context, where students face multiple academic, social, and personal pressures. In this sense, a lack of sleep could play a modulating role, amplifying the negative emotional impact of inappropriate social media use.

Although sleep disturbances partially mediated the relationship, explaining 31.4% of the total effect, a substantial proportion (68.6%) remains unexplained, suggesting the presence of additional mechanisms that should be explored in future studies. Previous evidence has identified several potential pathways, including increased exposure to social comparison and idealized content, which could negatively impact self-esteem and mood (Samra et al., 2022). Additionally, experiences such as online harassment or exposure to distressing content may contribute to depression independently of sleep (Alghamdi et al., 2025; Ye & Gao, 2025). Excessive social media use may also lead to social withdrawal or reduced face-to-face interaction, fostering feelings of isolation. Finally, maladaptive emotional regulation strategies, such as using social media to escape from negative emotions, may reinforce depressive symptoms over time. These alternative pathways warrant further investigation in future studies using comprehensive models that integrate psychological, behavioral, and contextual factors.

In addition to these behavioral and cognitive mechanisms, the partial mediation observed could also reflect a conceptual overlap between sleep disturbances and depressive symptomatology. Difficulty initiating or maintaining sleep is not only a diagnostic criterion for depression but also a factor that functionally disrupts emotion regulation, increases cognitive rumination, and heightens stress reactivity (Jansson-Fröjmark & Hossain, 2024; W. Wang et al., 2024). These psychological processes can amplify negative mood and reduce resilience to daily stressors, thereby reinforcing the depressive pathway. Importantly, in our study, sleep quality and depression were assessed using independent, validated instruments that capture distinct but interconnected domains (Pace-Schott et al., 2023). Therefore, the mediating effect of sleep disturbances likely represents both shared pathophysiological mechanisms and functional psychological processes through which inadequate sleep contributes to the onset and persistence of depressive symptoms.

To verify the stability of these findings, a sensitivity analysis was conducted using an alternative definition of social media addiction, in which the categories none and mild addiction were combined and compared with moderate and severe addiction. The results remained consistent with those of the main model, confirming that sleep disturbance continued to play a mediating role in the relationship between social media addiction and depressive symptoms. However, the proportion of the total effect explained through this pathway was smaller, which may reflect the greater predominance of direct mechanisms among individuals with more severe levels of social media use. These results support the robustness of our findings and highlight the complexity of the pathways linking problematic social media use to mental health outcomes.

### Limitations and Strengths

This study has some limitations that should be acknowledged. First, the cross-sectional design of our study does not allow for establishing a temporal sequence between social media addiction and sleep disturbances or depressive symptoms; therefore, the associations we estimate are not causal and should be interpreted with caution. Second, the variables were assessed using questionnaires; thus, participants’ data on social media use, sleep quality, or depressive symptoms could have been underestimated or overestimated. Furthermore, although validated instruments were used, they assess perceived symptoms or behaviors and do not replace clinical diagnostic evaluations. Nevertheless, this approach is common in epidemiological studies. Third, although the study included participants from three public universities in different Mexican states, which enhances representativeness within nursing programs, the findings may not be generalizable to students in private institutions, other academic disciplines, or other cultural contexts. However, the associations identified in this study are consistent with well-established biological mechanisms that link excessive screen exposure with sleep disturbance and depression. These mechanisms are not culturally bound, which supports the potential external validity of our findings beyond the studied population.

Despite the limitations above, this study has significant strengths. It is one of the first multicenter studies in Mexico to jointly analyze social media addiction, sleep quality, and depressive symptoms in nursing students, a population particularly vulnerable to academic, social, and emotional stressors. Since validated tests were used in the Mexican population and were administered by trained personnel, it is unlikely that our findings were affected by differential misclassification bias.

Additionally, the sample size was large and statistically justified, and the stratified sampling method adequately represented students from three public institutions in different regions of the country. Finally, the statistical models were adjusted for confounding variables, using DAGs and statistical criteria for identification, enhancing the robustness and internal validity of the findings.

## 5. Conclusions

Problematic social media use was associated with depressive symptoms among Mexican undergraduate nursing students, and the findings suggest that sleep disturbances may partially mediate this relationship. Our results highlight the importance of addressing problematic social media use as an emerging psychosocial risk factor in the university setting. They underscore the importance of implementing preventive strategies in academic nursing programs that foster healthy, critical use of digital technologies, promote balanced digital habits, and actively safeguard students’ mental health.

From a practical perspective, these findings pave the way for educational interventions, workshops, and institutional policies that integrate digital literacy, emotional self-care, and sleep hygiene as essential components of nursing staff training. Furthermore, the results can guide the design of psychoeducational support programs that identify and address risky digital behaviors and their consequences early. At the institutional level, nursing schools must recognize the impact of the digital environment on mental health and develop comprehensive student wellness policies that address the academic, technological, and psycho-emotional factors influencing professional performance during training.

While the mechanisms linking social media use, sleep disturbance, and depression are biologically universal, their contextual expression may differ across educational and cultural settings. By confirming this mediational pattern among Mexican nursing students using a counterfactual framework, our findings strengthen the external validity of previous evidence and highlight the need for culturally responsive strategies to promote mental well-being among healthcare students.

## Figures and Tables

**Table 1:** Sociodemographic, academic, and behavioral characteristics of undergraduate nursing students according to sleep disturbance and depressive symptoms.

Features	Total (n = 638)	Sleep Disturbance			Depression		
		No (n = 308)	Yes (n = 330)	*p*-Value ^a^	No (n = 462)	Yes (n = 176)	*p*-Value ^a^
	f (%)	f (%)	f (%)		f (%)	f (%)	
Sex							
Woman	516 (80.8)	244 (47.3)	272 (52.7)	0.304	380 (73.6)	136 (26.4)	0.153
Man	122 (19.1)	64 (52.5)	58 (47.5)		82 (67.2)	40 (32.8)	
Age							
18–20 years	414 (64.9)	191 (46.1)	223 (53.9)	0.287	290 (70.1)	124 (29.9)	0.138
21–23 years	185 (29.0)	95 (51.4)	90 (48.6)		140 (75.7)	45 (24.3)	
≥24 years	39 (6.1)	22 (56.4)	17 (43.6)		32 (82.1)	7 (17.9)	
Family history of mental illness							
Yes	101 (7.2)	43 (42.6)	58 (57.4)	0.211	57 (56.4)	44 (43.6)	0.001
No	537 (92.8)	265 (49.4)	272 (50.6)		405 (75.4)	132 (24.6)	
Study site							
Durango	280 (43.9)	191 (46.1)	223 (53.9)	0.287	196 (70.0)	84 (30.0)	0.129
Hidalgo	286 (44.8)	95 (51.3)	90 (48.7)		207 (72.4)	79 (27.6)	
Mexico City	72 (11.3)	22 (56.4)	17 (43.6)		59 (81.9)	13 (18.1)	
Marital status							
No partner	601 (94.2)	289 (48.1)	312 (51.9)	0.700	433 (72.1)	168 (27.9)	0.403
With partner	37 (5.8)	19 (51.4)	18 (48.6)		29 (78.4)	8 (21.6)	
Family monthly income							
<470 American dollars	373 (58.6)	169 (45.3)	204 (54.7)	0.075	259 (69.4)	114 (30.6)	0.046
≥470 American dollars	265 (41.5)	139 (52.5)	126 (47.5)		203 (76.6)	62 (23.4)	
Offspring							
Yes	46 (7.2)	13 (28.3)	33 (71.7)	0.005	32 (69.6)	14 (30.4)	0.654
No	592 (92.8)	295 (49.8)	297 (50.2)		430 (72.6)	162 (27.4)	
Year in program							
First	175 (27.4)	84 (48.0)	91 (52.0)	0.550	133 (76.0)	42 (24.0)	0.419
Second	187 (29.3)	94 (50.3)	93 (49.7)		132 (70.6)	55 (29.4)	
Third	164 (25.7)	72 (43.9)	92 (56.1)		113 (68.9)	51 (31.1)	
Fourth	112 (17.6)	58 (51.8)	54 (48.2)		84 (75.0)	28 (25.0)	
Scholarship							
Yes	29 (4.5)	13 (44.8)	16 (55.2)	0.704	18 (62.1)	11 (37.9)	0.202
No	609 (95.5)	295 (48.4)	314 (51.6)		444 (72.9)	165 (27.1)	
Employment							
Yes	162 (25.4)	86 (53.1)	76 (46.9)	0.156	40 (24.7)	122 (75.3)	0.340
No	476 (74.6)	222 (46.6)	254 (53.4)		340 (71.4)	136 (28.6)	
Mother’s education							
Primary education	76 (11.9)	32 (42.1)	44 (57.9)	0.139	49 (64.5)	24 (35.5)	0.028
Lower secondary education	271 (42.5)	123 (45.4)	148 (54.6)		190 (70.1)	81 (29.9)	
Upper secondary education	195 (30.6)	98 (50.3)	97 (49.7)		144 (73.8)	51 (26.2)	
Tertiary education	96 (15.0)	55 (57.3)	41 (42.7)		80 (83.3)	16 (16.7)	
Father’s education							
Primary education	54 (8.5)	26 (48.2)	28 (51.8)	0.849	36 (66.7)	18 (33.3)	0.269
Lower secondary education	271 (42.5)	130 (48.0)	141 (52.0)		189 (69.7)	82 (30.3)	
Upper secondary education	185 (29.0)	86 (46.5)	99 (53.5)		141 (76.2)	44 (23.8)	
Tertiary education	128 (20.0)	66 (51.6)	62 (48.4)		97 (75.8)	31 (24.2)	
Regular alcohol consumption							
Yes	77 (12.1)	45 (58.4)	32 (41.6)	0.057	54 (70.2)	23 (29.8)	0.609
No	561 (87.9)	263 (46.9)	298 (53.1)		409 (72.9)	152 (27.1)	
Regular cigarette smoking							
Yes	65 (10.2)	37 (56.9)	28 (43.1)	0.141	46 (70.7)	19 (29.3)	0.731
No	573 (89.8)	271 (47.3)	302 (52.7)		417 (72.7)	156 (27.3)	
Hours on social media per day							
≤2 h	135 (21.2)	82 (60.7)	53 (39.3)	0.003	112 (52.3)	23 (17.0)	<0.001
3–5 h	287 (45.0)	133 (46.3)	154(53.7)		217 (75.6)	70 (24.4)	
6–8 h	160 (25.0)	74 (46.2)	86(53.8)		104 (65.0)	56 (35.0)	
≥9 h	56 (8.8)	19 (33.9)	37(66.1)		30 (53.6)	26 (46.4)	

Abbreviations: f, frequency. ^a^ Comparing subjects by sleep disturbance and depression status using Pearson’s chi-squared test.

**Table 2:** Sleep disturbance and depressive symptoms by levels of social media addiction.

Social Media Addiction	Total	Sleep Disturbance			Depressive Symptoms		
		No	Yes	*p*-Value ^a^	No	Yes	*p*-Value ^a^
	f (%)	f (%)	f (%)	f (%)	f (%)	f (%)	f (%)
No addiction	125 (19.6)	79 (63.2)	46 (36.8)	<0.001	104 (83.2)	21 (16.8)	<0.001
Mild dependence	205 (32.1)	115 (56.1)	90 (43.9)		171 (83.4)	34 (16.6)	
Moderate dependence	175 (27.4)	73 (41.7)	102 (58.2)		113(64.6)	62 (35.4)	
High dependency	133 (20.9)	41 (30.8)	92 (69.2)		74 (55.6)	59 (44.4)	

Abbreviations: f, frequency. ^a^ Comparing subjects by depression and sleep disturbance status using Pearson’s chi-squared test.

**Table 3:** Raw and adjusted odds ratios of the relationship between problematic social media use and sleep disturbance.

Problematic Social Media Use	Sleep Disturbance			
	OR (95% IC)	*p*-Value	OR ^a^ (95% CI)	*p*-Value
No addiction	Ref.		Ref.	
Mild dependence	1.34 (0.85, 2.12)	0.204	1.31 (0.81, 2.10)	0.263
Moderate dependence	2.39 (1.49, 3.84)	<0.001	2.22 (1.37, 3.61)	0.001
High dependency	3.85 (2.29, 6.49)	<0.001	3.73 (2.19, 6.35)	<0.001
*p*-trend	<0.001		<0.001	

Abbreviations: OR, odds ratio; CI, Confidence interval. ^a^ Adjusted for age, sex, year in program, paid work, family monthly income, study location, family history of mental illness, and parental education.

**Table 4:** Raw and adjusted odds ratios of the relationship between problematic social media use and depressive symptoms.

Problematic Social Media Use	Depressive Symptoms			
	OR (95% CI)	*p*-Value	OR ^a^ (95% CI)	*p*-Value
No addiction	Ref.		Ref.	
Mild dependence	0.98 (0.54, 1.78)	0.960	1.05 (0.63, 2.09)	0.872
Moderate dependence	2.71 (1.54, 4.76)	<0.001	2.56 (1.41, 4.64)	0.002
High dependency	3.94 (2.20, 7.05)	<0.001	4.54 (2.45, 8.44)	<0.001
	<0.001		<0.001	

Abbreviations: OR, odds ratio; CI, Confidence interval. ^a^ Adjusted for age, sex, year in program, paid work, family monthly income, study location, family history of mental illness, and parental education.

## Data Availability

The data that support the findings of this study are openly available in Mendeley Data at doi: 10.17632/y478b26pnc.1; https://data.mendeley.com/datasets/y478b26pnc/1 (accessed on 11 August 2025).

## Supplementary Materials

The following supporting information can be downloaded at: https://www.mdpi.com/article/10.3390/ejihpe15110229/s1, Supplementary Table S1. Distribution of the Study Sample by Recruitment Site Based on Proportional Stratified Sampling; Supplementary Figure S1: Directed acyclic graph for the hypothesized counterfactual mediation structure in which social media addiction influences depressive symptoms directly and indirectly through sleep disturbance; Supplementary Figure S2: Sensitivity analysis of the mediation model.

## Abbreviations

The following abbreviations are used in this manuscript:

CESD-7	Center for Epidemiologic Studies Depression Scale
SMAS-SF	Social Media Addiction Scale-Student Form
PSQI	Pittsburgh Sleep Quality Index
OR	Odds ratio
CI	Confidence interval
DAGs	Directed acyclic graphs
