# Supplementary material for: Sleep Disturbance as a Mediator Between Problematic Social Media Use and Depressive Symptoms Among Mexican Undergraduate Nursing Students: A Multicenter Study
Source: Eur J Investig Health Psychol Educ. 2025 Nov 11;15(11):229. doi: 10.3390/ejihpe15110229 (PMC12651063; doi:10.3390/ejihpe15110229)
Supplement: Supplementary file 1 [file ejihpe-15-00229-s001.zip › ejihpe-3906773-supplementary.pdf]

Supplementary Figure S1.

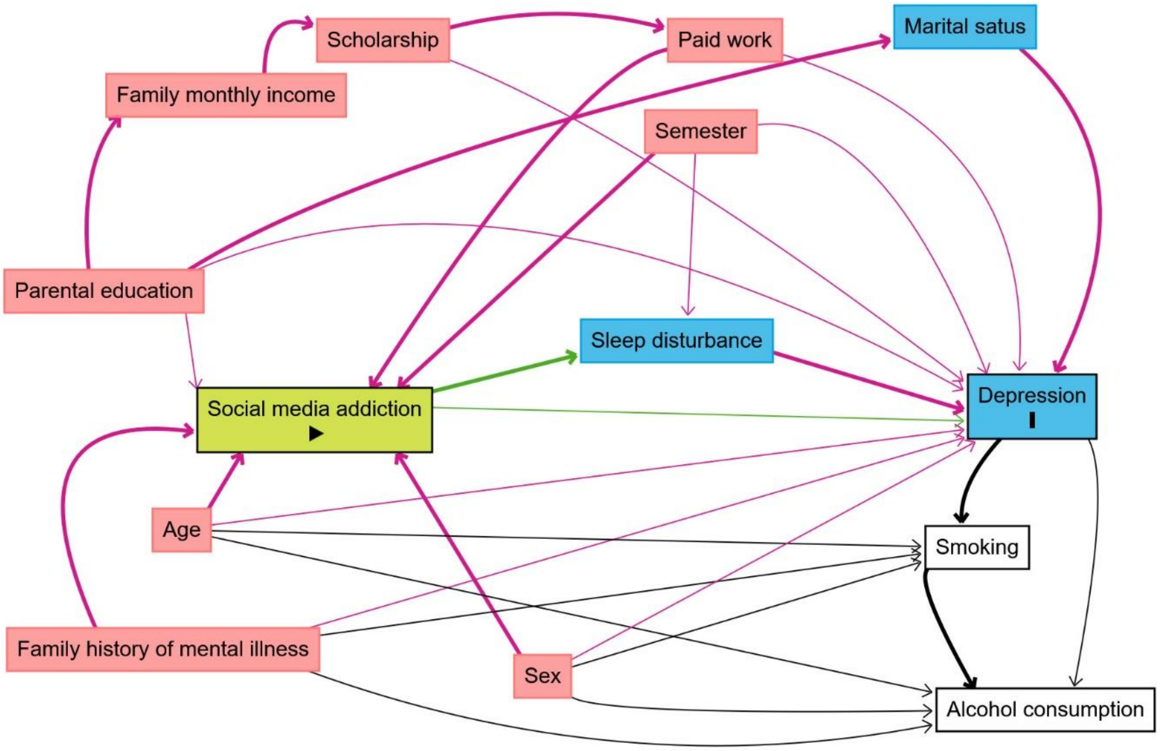

Figure S1. DAG illustrating the hypothesized counterfactual mediation structure in which social media addiction influences depressive symptoms directly and indirectly through sleep disturbance

Supplementary Figure S2: Sensitivity analysis of the mediation model

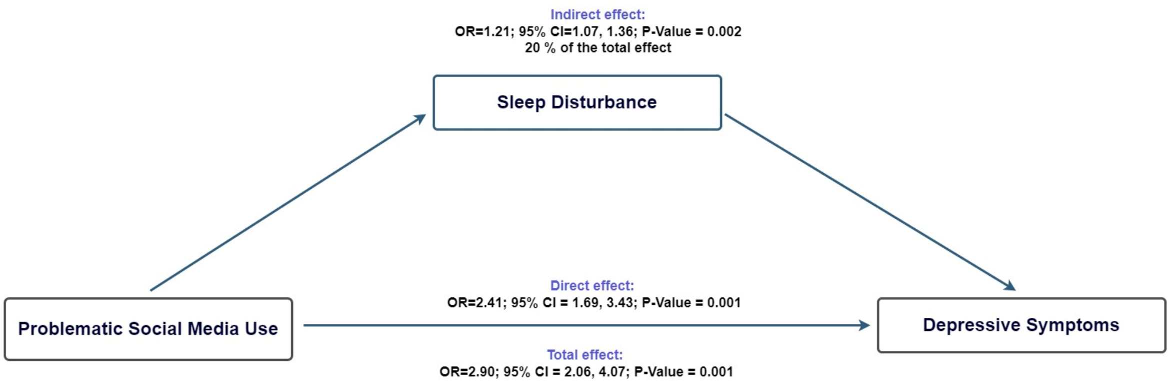

Sensitivity analysis of the mediation model showing the direct, indirect, and total effects of problematic social media use on depressive symptoms through sleep disturbance. For this analysis, the no-dependence and mild-dependence categories were collapsed and compared with the moderate- and high-dependence levels.

Supplementary Table S1. Distribution of the Study Sample by Recruitment Site Based on Proportional Stratified Sampling (sample size=638)

Site location	Population size in each stratum (a)	Proportion of the stratum relative to the total (b)	Sample size allocated proportionally to each stratum (c)
Durango	597	0.438	280
Mexico City	154	0.113	72
Hidalgo	610	0.448	286
Total	1361	1.000	638

b= a/total

c= b/total * sample size
